# Convergence of virulence and resistance in international clones of WHO critical priority enterobacterales isolated from Marine Bivalves

**DOI:** 10.1038/s41598-022-09598-8

**Published:** 2022-04-05

**Authors:** Vanessa Bueris, Fábio P. Sellera, Bruna Fuga, Elder Sano, Marcelo P. N. Carvalho, Samuel C. F. Couto, Quézia Moura, Nilton Lincopan

**Affiliations:** 1 Department of Microbiology, Institute of Biomedical Sciences, University of São Paulo, São Paulo, Brazil; 2 Laboratory of Genetics, Butantan Institute, São Paulo, Brazil; 3 Department of Internal Medicine, School of Veterinary Medicine and Animal Science, University of São Paulo, São Paulo, Brazil; 4 School of Veterinary Medicine, Metropolitan University of Santos, Santos, Brazil; 5 One Health Brazilian Resistance Project (OneBR), São Paulo, Brazil; 6 Department of Clinical Analysis, Faculty of Pharmaceutical Sciences, University of São Paulo, São Paulo, Brazil; 7 Department of Veterinary Clinic and Surgery, Federal University of Minas Gerais, Belo Horizonte, MG Brazil

**Keywords:** Antimicrobial resistance, Food microbiology

## Abstract

The global spread of critical-priority antimicrobial-resistant Enterobacterales by food is a public health problem. Wild-caught seafood are broadly consumed worldwide, but exposure to land-based pollution can favor their contamination by clinically relevant antimicrobial-resistant bacteria. As part of the Grand Challenges Explorations: New Approaches to Characterize the Global Burden of Antimicrobial Resistance Program, we performed genomic surveillance and cell culture-based virulence investigation of WHO critical priority Enterobacterales isolated from marine bivalves collected in the Atlantic Coast of South America. Broad-spectrum cephalosporin-resistant *Klebsiella pneumoniae* and *Escherichia coli* isolates were recovered from eight distinct geographical locations. These strains harbored *bla*_CTX-M_-type or *bla*_CMY_-type genes. Most of the surveyed genomes confirmed the convergence of wide virulome and resistome (i.e., antimicrobials, heavy metals, biocides, and pesticides resistance). We identified strains belonging to the international high-risk clones *K. pneumoniae* ST307 and *E. coli* ST131 carrying important virulence genes, whereas in vitro experiments confirmed the high virulence potential of these strains. Thermolabile and thermostable toxins were identified in some strains, and all of them were biofilm producers. These data point to an alarming presence of resistance and virulence genes in marine environments, which may favor horizontal gene transfer and the spread of these traits to other bacterial species.

## Introduction

The rapid and global dissemination of critical-priority antimicrobial-resistant Enterobacterales is a public health problem that demands mitigation strategies and strengthening by genomic epidemiological surveillance investigations^1^. Given its epidemiological importance, there are increasing reports of critical-priority bacteria in aquatic environments^2^, including coastal waters^3–5^, reinforcing that polluted marine environments may act as potential reservoirs for waterborne pathogens.

Seafood consumption has increased significantly during the last decades^6^. Although domestic aquaculture farming has also presented a linear trend of increase, wild-caught seafood remains broadly consumed globally^7–9^. In this regard, marine bivalves have a wide geographic distribution, being considered an alternative food protein source to coastal populations worldwide since ancient times^10^. They have also been used as marine sentinels and investigated as potential ecological bioindicators to measure environmental impacts related to water pollution due to their activity in filtering particles suspended in the surrounding water^11^.

Recent studies have documented human gastroenteritis outbreaks associated with the consumption of contaminated bivalves^12–14^. In this regard, norovirus and *Vibrio* spp. infections have gathered scientific community attention due to the pathogenicity and virulence behavior of these etiologic agents^13–15^. Although some studies have speculated that the identification of multidrug-resistant (MDR) bacteria in marine bivalves could also represent a potential threat for seafood consumers^12,16,17^, there is scarce scientific evidence regarding this matter.

Here, we report the emergence of critical priority Enterobacterales producing CTX-M-type and CMY-type β-lactamases, and thermolabile and thermostable toxins in wild-caught marine bivalves in the Southeast coast of Brazil, South America's largest and most populated country. We performed a robust investigation using WGS and in vitro experiments to assay the virulence potential of these bacteria to infect human cells, highlighting a new and hidden threat for seafood consumers.

## Results

### Occurrence of multidrug-resistant clinical relevant bacteria in edible marine bivalves

**Figure 1:**
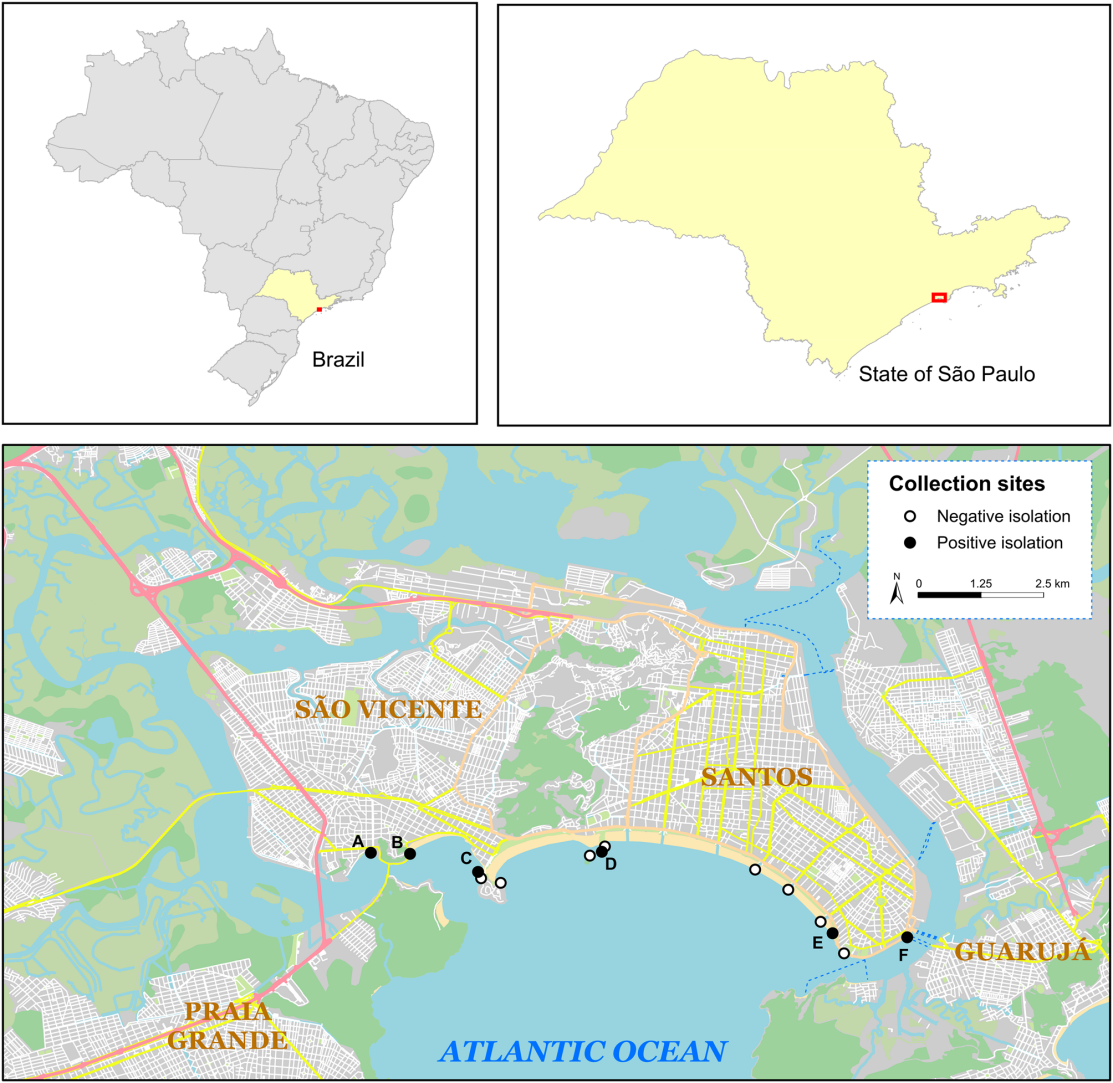
Map showing 14 different collection sites on the coastal areas of São Vicente and Santos cities, Southeast Brazil. Critical priority Enterobacterales strains were isolated from bivalves at 6 of these sites (represented by black markers): (**A**)—*E. coli* TM2CRO [location, −23.972588 S, −46.391932 W]; (**B**)—*K. pneumoniae* JM2CRO [location, −23.972765 S, −46.384877 W]; (**C**)—*E. coli* MM and *E. coli* MO [location, −23.976040 S, −46.372580 W]; (**D**)—*E. coli* EM1CRO [location, −23.972388 S, −46.350241 W]; (**E**)—*E. coli* C6O and *E. coli* 6 M [location, −23.987125 S, −46.308609 W]; (**F**)—*K. pneumoniae* BO2 [location, −23.987841 S, −46.295134 W]). The map was generated using public domain shapefiles provided by the Brazilian Institute of Geography and Statistics (https://www.ibge.gov.br), and OpenStreetMap vector layers (©OpenStreetMap contributors), licensed under the Open Data Commons Open Database License (https://www.openstreetmap.org/copyright). OpenStreetMap vector layers were downloaded on QGIS v3.22.4 (https://qgis.org) with OSMDownloader plugin v1.0.3 (https://github.com/lcoandrade/OSMDownloader). Final layout was designed on ESRI ArcMap™ v10.7.

**Figure 2:**
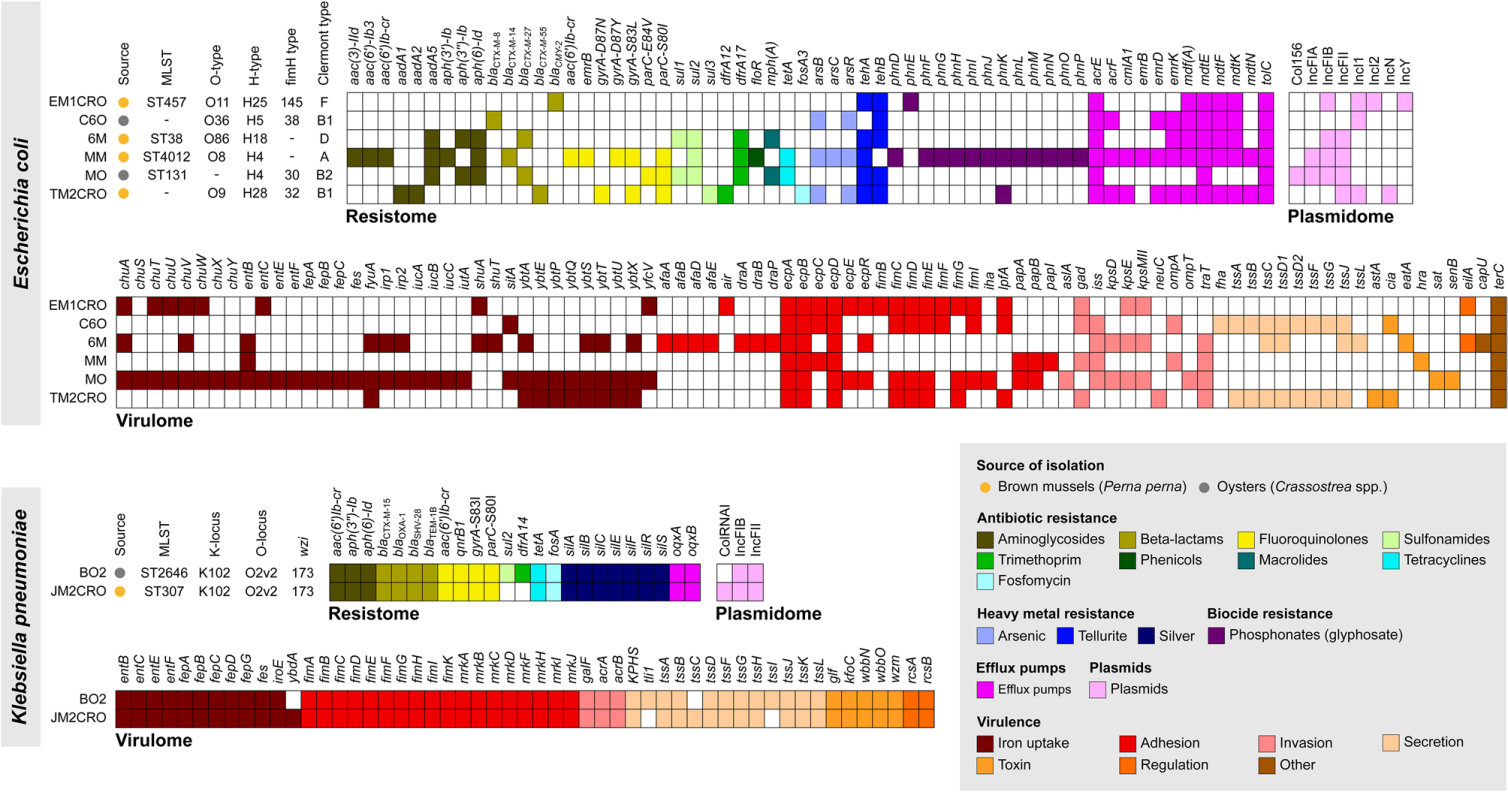
Heatmap of virulome, resistome, plasmidome, MLST and serotype of critical priority WHO Enterobacterales isolated from wild edible marine bivalves recovered from anthropogenically-polluted area on the South America Atlantic coast.

Eight ceftriaxone-resistant Enterobacterales, including *E. coli* (*n* = 6) and *K. pneumoniae* (*n* = 2) isolates, were recovered from edible marine bivalves collected in different points on the southeast coast of Brazil (Fig. 1 and Table 1). Four *E. coli* and one *K. pneumoniae* were found in brown mussels, whereas two *E. coli* and one *K. pneumoniae* were obtained from oysters. All isolates displayed a MDR resistance profile, but still remained susceptible to carbapenems and colistin (Fig. 2). Seven isolates exhibited an ESBL phenotype, presenting cefotaxime MIC > 32 µg/mL. Additionally, one *E. coli* (EM1CRO) was cefoxitin-resistant (MIC > 32 µg/mL).

**Table 1:** Epidemiological characteristics of critical priority WHO Enterobacterales strains isolated from edible marine bivalves.

Strain	Bivalve species	Location	ST/CC*	Serotype	Accession number
*E*. *coli* EM1CRO	Brown mussels (*Perna perna*)	−23.972388 S, −46.350241 W	457/-	O11:H25	NDBC00000000.1
*E*. *coli* C6O	Oysters (*Crassostrea* spp.)	−23.987125 S, −46.308609 W	ND	O36:H5	NDBB00000000.1
*E*. *coli* 6 M	Brown mussels (*Perna perna*)	−23.987125 S, −46.308609 W	38/38	O86:H18	NCWA00000000.1
*E*. *coli* MM	Brown mussels (*Perna perna*)	−23.976040 S, −46.372580 W	4012/-	O8:H4	NDYX00000000.1
*E*. *coli* MO	Oysters (*Crassostrea* spp.)	−23.976040 S, −46.372580 W	131/131	-:H4	NCVZ00000000.1
*E*. *coli* TM2CRO	Brown mussels (*Perna perna*)	−23.972588 S, −46.391932 W	ND	O9:H28	NCVY00000000.1
*K*. *pneumoniae* BO2	Oysters (*Crassostrea* spp.)	−23.987841 S, −46.295134 W	2646/-	O2v2:K102	NCVX00000000.1
*K*. *pneumoniae* JM2CRO	Brown mussels (*Perna perna*)	−23.972765 S, −46.384877 W	307/-	O2v2:K102	NCVW00000000.1

* CC, clonal complex; ST, sequence type; ND, not determined.

### Genomic analyses reveal the convergence of broad virulome and resistome in seafood bacterial isolates

Resistome data of *E. coli* strains EM1CRO, MO, 6M were previously reported under an epidemiological context^18,19^. In the current study, we carried out an in-depth genomic analysis that also included new *E. coli* C60, MM and TM2CRO and *K. pneumoniae* BO2 and JM2CRO strains. The main results from WGS analysis are presented in Fig. 2. The convergence of wide virulome and resistome (antimicrobials, heavy metals, biocides, and pesticides resistance genes) was confirmed in most of the surveyed genomes. In this regard, all strains were positive to CTX-M variants, including *bla*_CTX-M-8_ (C6O), *bla*_CTX-M-14_ (MM), *bla*_CTX-M-15_ (BO2 and JM2CRO), *bla*_CTX-M-27_ (MO and 6M), and *bla*_CTX-M-55_ (TM2CRO), except for *E. coli* EM1CRO, which was positive for the plasmid-mediated AmpC (pAmpC) gene, *bla*_CMY-2._ Unfortunately, since we used a short read sequencing technology, we were not able to assemble the plasmids completely to verify all genes and their respective plasmid replicons. However, through mlplasmids tool (https://sarredondo.shinyapps.io/mlplasmids/) it was possible to predict the genes found in plasmids for *E. coli* strains (EM1CRO: *mdfA*; 6M: *bla*_CTX-M-27_; MO: *aadA5*, *sul1*, *dfrA17*, *mphA*; and TM2CRO: *bla*_CTX-M-55_).

A broad virulence repertoire was found in most strains, where lineages of phylogroups B2 (MO) and D (6M) exhibited a wider virulome than those of phylogroups A (MM), B1 (C6O and TM2CRO), and F (EM1CRO). MDR strains belonged to different STs, and worryingly an *E. coli* strain of ST131 (MO) and a *K. pneumoniae* strain of ST307 (JM2CRO) were detected. Overall, *E. coli* strains exhibited a large number of genes associated with adhesion to the host (i.e.,* iha*, *lpf*, *pap*, *ecp*, *fim*, and *afa*/*dr fimbriae*), resistance to the host’s immune system (i.e.,* kps* and *neuC* capsular polysaccharides; *tra* and *iss* complement and serum resistance), damage to the host (i.e.,* senB* and *astA* enterotoxins) and iron/hemin uptake (i.e.,* chu*, *iuc*, *ent*, *irp*, and *ybt*). The serotype and *fimbriae* type of *E. coli* strains were diverse and included O11:H25, O36:H5, O86:H18, O8:H4, and O9:H28; *fimH145*, *fimH38*, *fimH30*, and *fimH32*. *K. pneumoniae* strains also exhibited genes associated with adhesion to the host (i.e.,* fim* operon), resistance to the host’s immune system (i.e.,* kfoC* complement and serum resistance), damage to the host and iron/hemin uptake (i.e.,* iro* and *ent*). We also identified the K-locus KL102/wzi173 in both BO2 and JM2CRO *K. pneumoniae* strains. Genome assembly revealed that IncF-type plasmids were the most prevalent among the strains. The limitations of short-read sequencing allowed only the type of plasmid (IncFII) associated with the *bla*_CTX-M_ gene to be determined in the *K. pneumoniae* BO2 strain.

### Virulent behavior of Enterobacterales isolated from edible marine bivalves

**Figure 3:**
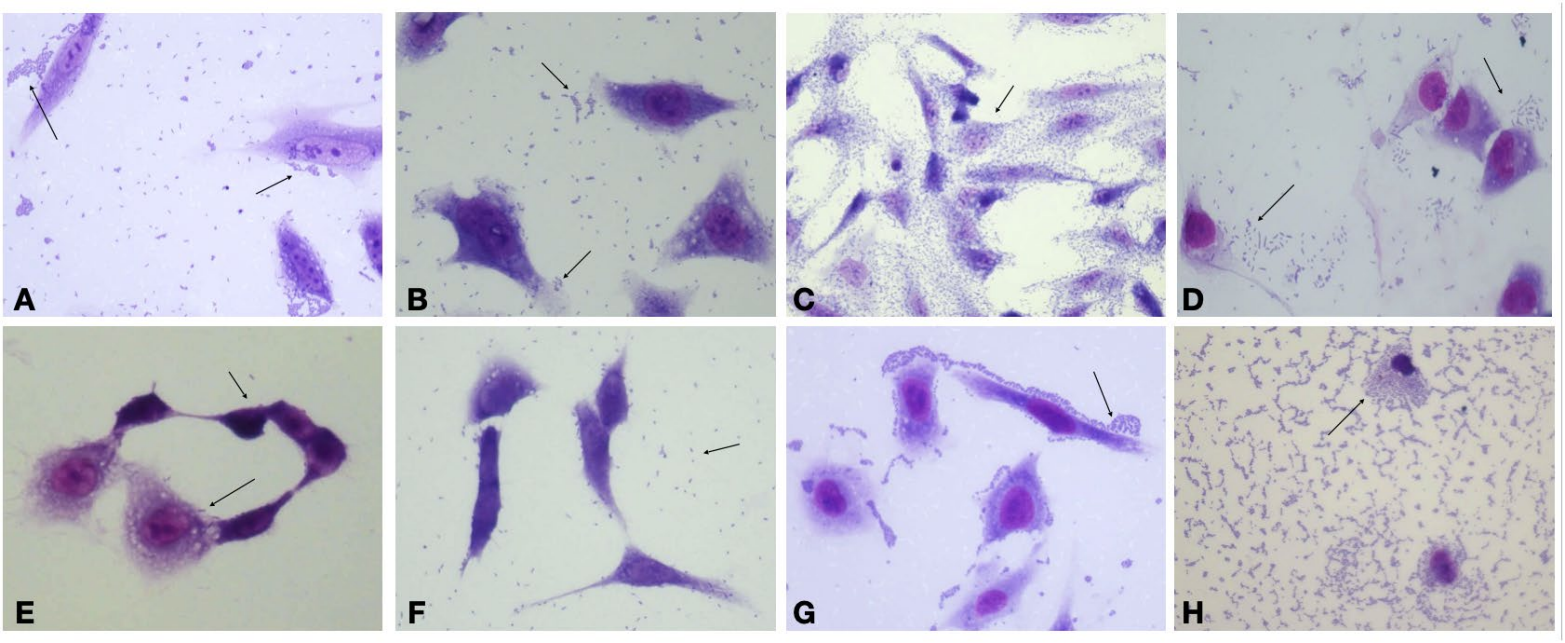
In vitro adhesion patterns displayed by the Enterobacterales strains isolated from edible marine bivalves, after 6 h incubation with HeLa cells. *E.coli* strain EM1CRO O11:H25 (**A**), *E. coli* strain C6O O36:H5 (**B**), *E. coli* 6 M O86:H18 (**C**), *E. coli* MM O8:H4 (**D**), *E. coli* MO O25:H4 (**E**), *E. coli* TM2CRO O9:H28 (**F**), *K. pneumoniae* BO2 (**G**) and *K. pneumoniae* JM2CRO (**H**). Arrows point to the adhered bacteria. Magnification 400x.

**Figure 4:**
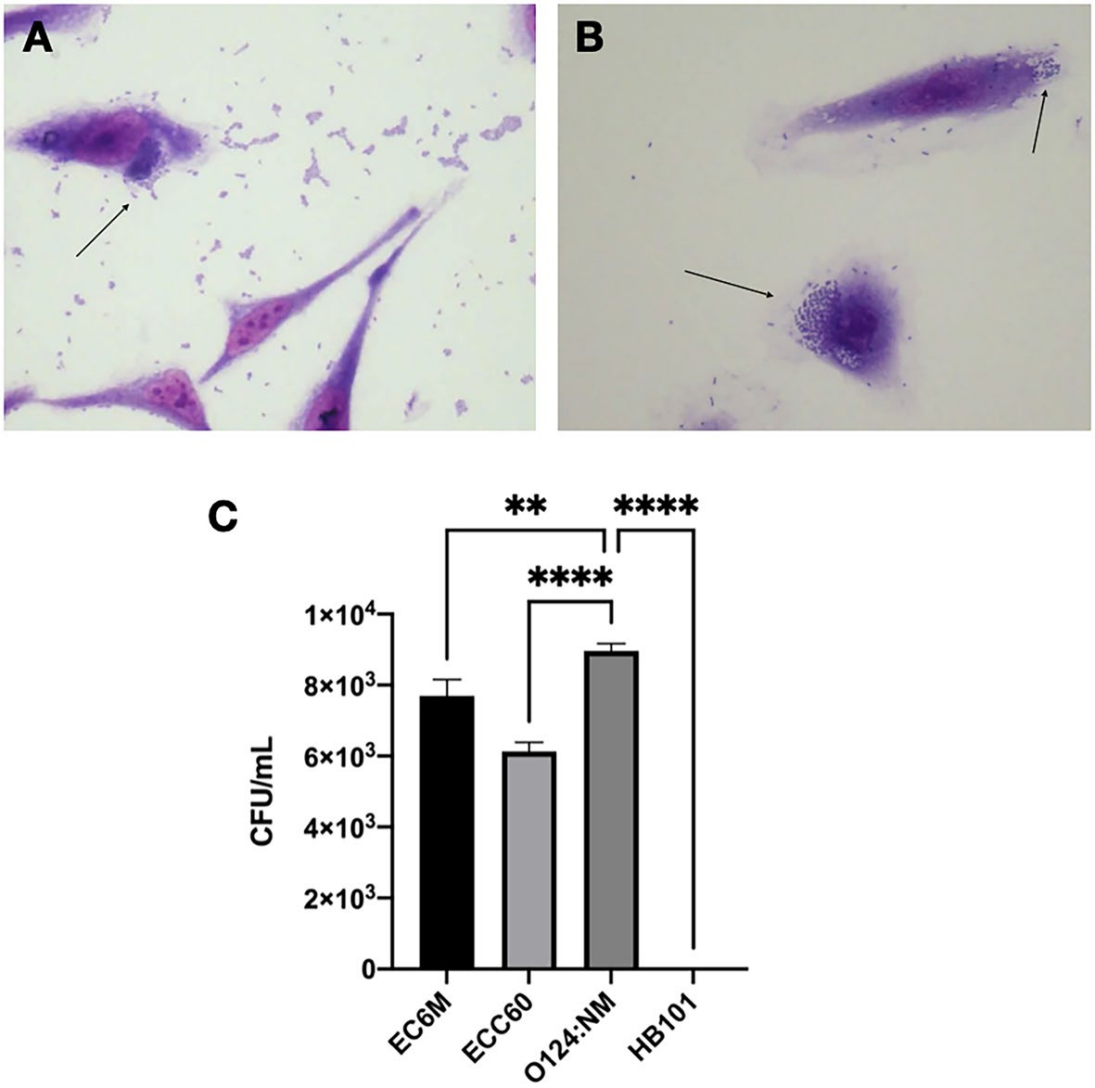
Invading phenotype exhibited in vitro by *E. coli* strains C6O O36:H5 (**A**) and 6 M O86:H18 (**B**). Arrows point to the invasion sites. Magnification 400x. The number of invading bacteria was determined by the invasion assay (**C**) and compared to 0124:NM (positive control) and HB101 (negative control) strains; ** < 0.01 **** < 0,0001.

Bacterial isolates were tested to verify their ability to colonize and damage human epithelial cells. In this regard, all *E. coli* isolates were capable of adhering to HeLa cells after 6 h of incubation (Fig. 3), even though they displayed different adherence patterns with EM1CRO and C6O strains showing aggregative adherence, 6M and TM2CRO strains adhered diffusely to the cells, and MM and MO displaying an undefined adherence pattern. The two *K. pneumoniae* strains also exhibited aggregative adherence, both on the surface of the epithelial cells and on the glass slide. Additionally, *E. coli* strains 6M and C6O were capable of invading HeLa Cells after 6 h of incubation (Fig. 4), as observed through microscopy and confirmed by the gentamicin protection (invasion) assay.

**Figure 5:**
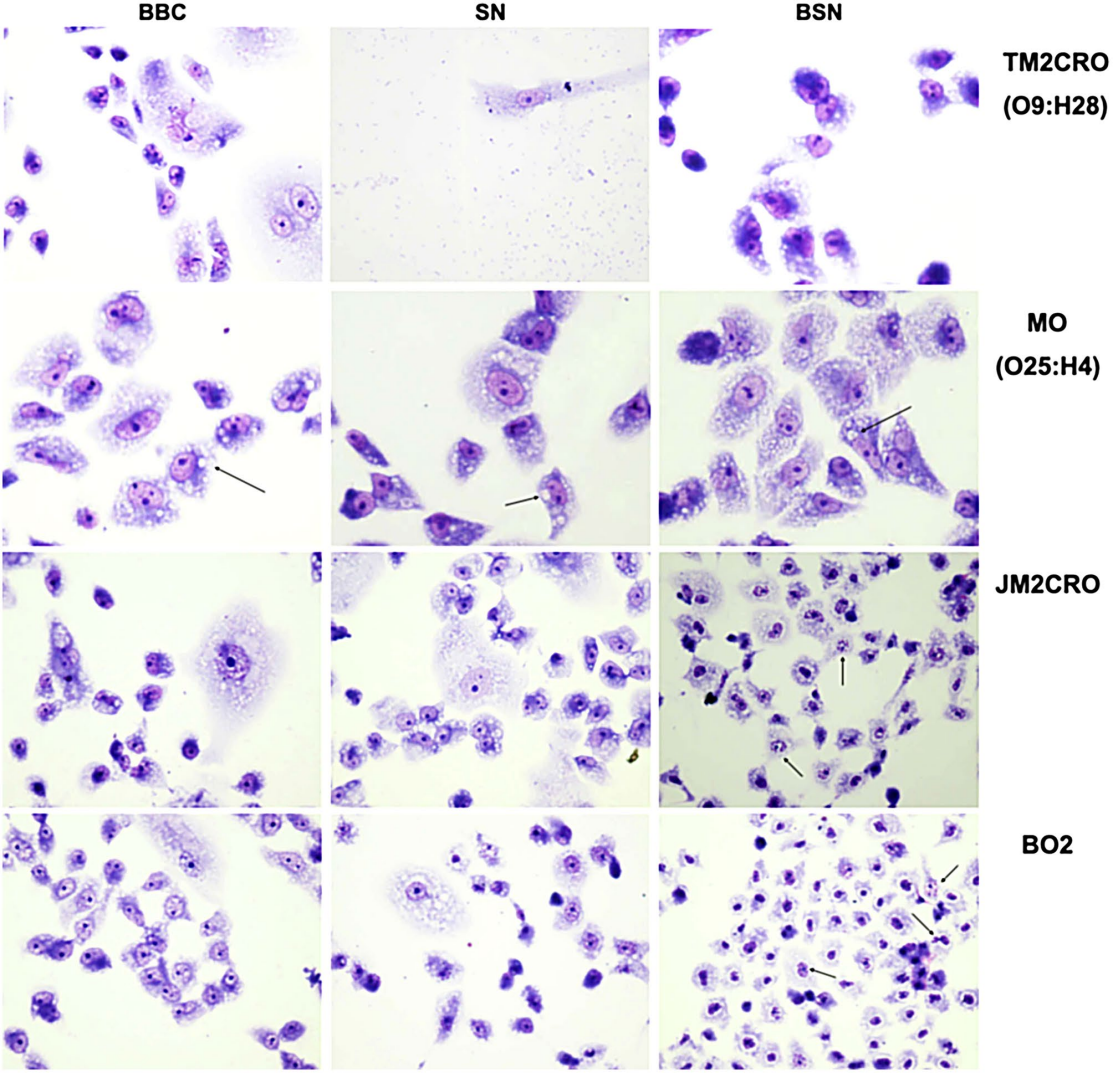
Cytotoxic assay on Vero cells. BBC, boiled bacterial cells; SN, supernatant; BSN, boiled supernatant. Arrows point to vacuollation and nuclear damage induced by *E. coli* MO (O25:H4), and *K. pneumoniae* JM2CRO and BO2 strains, respectively. Magnification 400x.

Once MO, TM2CRO, and JM2CRO strains were visually confirmed as toxic to HeLa cells, cytotoxic assays on Vero cells were also performed (Fig. 5). Interestingly, when incubated with live bacterial cells, all strains were toxic to Vero cells. To verify if this was caused by cell–cell contact, Vero cells were incubated with dead bacteria and no effect was observed. To establish if secreted proteins were involved in toxicity, filtered supernatant from bacterial strains were incubated with Vero cells and only the TM2CRO strain resulted in cell damage. In order to determine if any secreted toxins were thermostable, boiled, filtered supernatant was added to the cells. This result suggested that *E. coli* TM2CRO expresses a thermolabile toxin since no effect on cells was observed after following this treatment. Interestingly, a vacuolating effect was observed in all MO treatments, which could indicate that this strain expresses a thermostable secreted toxin. Regarding the *K. pneumoniae* strains, JM2CRO and BO2 boiled supernatants caused nuclear damage to Vero cells, indicating that these strains probably secrete a thermostable toxin.

**Figure 6:**
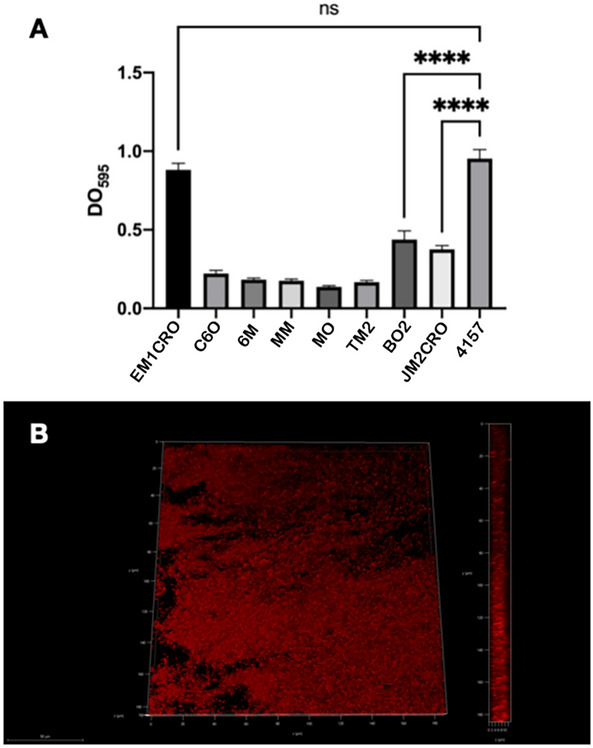
Biofilm formation by critical priority WHO Enterobacterales from isolated from wild edible marine bivalves. In **A**, crystal violet assay, after 24 h incubation at 37 °C in BHI medium. The values correspond to the average of three independent triplicates; *p* values < 0.001 were considered significant. Strains were considered to form weak, moderate, or strong biofilms, compared to a strong biofilm-forming *E. coli* strain (4157); In **B**, confocal laser scanning microscopy of EM1CRO O11:H25 biofilm on glass coverslips, after 24 h incubation at 37 °C in BHI medium and propidium iodide staining.

All strains evaluated in this study were able to form biofilms, although they were considered, in their majority, to form weak or moderate biofilms under the tested conditions (Fig. 6a). However, the EM1CRO strain was an exception since it formed a biofilm as robust as the *E. coli* 4157, considered to be a highly biofilm-forming control strain (Fig. 6b).

## Discussion

The presence of ESBL and/or pAmpC-positive *K. pneumoniae* and *E. coli* strains contaminating bivalves in 6 of 14 investigated geographical points suggests heavy contamination of the marine environment of this geographical region, with critical priority bacteria. Indeed, nearby cities have a history of marine pollution due to lack of sewage treatment and sanitation facilities^20^, with the occurrence of critical priority bacteria in their coastal waters, and additional contamination of wild-caught fishes being recently documented^21–25^. Therefore, the presence of human fecal pollution in these coastal waters can support the origin of bioaccumulation of such pathogens in marine bivalves since they are filter-feeding organisms that remove a large amount of suspended material from the water.

While *E. coli* is a genetically heterogeneous species that can cause both diarrheal diseases and MDR extra-intestinal infections, *K. pneumoniae* has been a significant cause of antimicrobial-resistant healthcare-associated infections. In addition, worldwide dissemination of both species has been associated with well-established evolutionary pathogenic lineages, such as *K. pneumoniae* ST307 and *E. coli* ST131, which often display an MDR phenotype and a combination of virulence genes^26,27^.

Worryingly, in our study, we identified the presence of international CTX-M-27-producing *E. coli* ST131 and CTX-M-15-producing *K. pneumoniae* ST307 clones in oysters and mussel samples, respectively. This should be viewed as an epidemiological alert, since several studies have reported the rapid spread of these high-risk MDR clones at the human-animal interface, with several outbreaks worldwide^28–30^. The spread of virulence and MDR profiles is directly associated with the harboring of mobile genetic elements, and among them, plasmids are considered to play an essential role. A direct association between high-risk strains and the presence of IncF plasmids has been demonstrated, especially those containing FIA and FII replicon types^31,32^, which were detected in the majority of bacteria isolated in this study.

Currently, *E. coli* ST131 has been the most prevalent ExPEC clone circulating globally, being composed of multiple strains with distinct resistance profiles^33^, which might indicate that its emergence is involved in different events related to horizontal transfer of antimicrobial resistance genes. Indeed, each ST131 subclone is associated with a specific allele of the type 1 fimbrial adhesin gene (*fimH*), being the *H*30 the most prevalent. Of note, *E. coli* ST131-*fimH*30 has been responsible for urinary tract infections worldwide, being frequently associated to fluoroquinolone resistance^34^. On the other hand, the subclone *H*22 has been associated with foodborne infections^35^. Although the *E. coli* ST131 identified in study belongs to subclone *H*30, it could also represent a possible threat for foodborne infections since it has already been isolated from food-producing animals, including retail chicken^36,37^. Interestingly, most of the strains in subclone *H*30 belong to serotype O25:H4. In this study, the *E. coli* ST131-*fimH*30 (MO strain) was identified as H4 type flagellum, which has characteristics that aid in host colonization processes and is also capable of triggering enhanced induction of the cytokine IL-10, contributing to its fitness in the urinary tract^38^.

To cause infection, microorganisms must be able to colonize their host and adherence is a crucial event to the colonization process. Bacterial pathogens usually express a variety of fimbrial and afimbrial adhesins that promote attachment to the host cells by interacting with surface receptors. For certain *E. coli* pathotypes, the presence of these structures may result in specific adhesion patterns on epithelial cells. EM1CRO and C6O strains exhibited an adhesion pattern known as aggregative. Enteroaggregative *E. coli* (EAEC), usually related to persistent diarrhea, often expresses AAF/I and AAF/II adhesins^39^, which were not detected by WGS. However, both strains harbor the *lpfA* gene that encodes the long polar fimbriae commonly found in EAEC^40^. Additionally, the presence of the *fim* operon, which encodes a type I fimbria, could explain the aggregative adhesion pattern observed in these two strains^41^. EM1CRO also harbors the *air* and *eilA* genes. Air is an adhesin that is under the control of *eilA* and promotes bacterial aggregation and colonization^42,43^, contributing to aggregative adherence. On the other hand, 6M and TM2CRO strains adhered diffusely to epithelial cells. Diffusely adherent *E. coli* (DAEC) comprise a heterogeneous group without specific genetic markers. Still, a large proportion of DAEC strains express Afa/Dr adhesins and are frequently involved in chronic diarrhea in children^44^. The genes encoding this adhesin were found only in the 6M strain.

MM and MO *E. coli* strains showed an undefined adhesion pattern. Both strains harbor the *E. coli* common pilus (*ecp*) operon, which mediates adherence to the host cell by pathogenic and commensal strains of *E. coli*^45^, and presented genes from the homologous *pap* operon, which encodes the P fimbriae. Interestingly, MM adhered preferably to the coverslips, while MO mainly adhered to epithelial cells. This difference could be due to the presence of the *iha* gene in the MO strain, which encodes a bacterial adherence-conferring protein that was described as sufficient to confer adherence phenotype in non-adherent *E. coli* strains^46^, and the presence of the *fim* operon.

Although not usually classified by adherence, *K. pneumoniae* BO2 and JM2CRO strains showed an aggregative adherence pattern. Genome sequencing detected the presence of genes that encode mannose-resistant *Klebsiella*-like hemagglutinins (Mrk) proteins in both strains. These proteins compose a type 3 fimbriae that participates in biofilm formation, and can result in the establishment of bacterial aggregates in biotic and abiotic surfaces^47^. In addition, these strains also have the *fim* operon, which encodes the type 1 fimbriae.

Bacterial attachment to host cells usually triggers host signal transduction cascades, which may induce the internalization of bacterial cells due to the consequent rearrangement of eukaryotic cytoskeleton components^48^. Bacterial pathogens capable of invading host cells and tissues can successfully evade host defenses and spread easily. Interestingly, 6M and C6O strains presented the cell invasion phenotype, although no gene encoding known *E. coli* invasins was detected. It is important to note that the 6M and MO strains met the requirements to be classified as ExPEC^49^, belonging to phylogroups D and B2, respectively, which are reported to show higher virulence in humans^50^. ExPEC lacks pathogenicity when it colonizes the host's intestine, but it can cause potentially fatal diseases when it is transferred to other sites in the body. These strains differ widely in their genetic content and have an extensive range of virulence factors that assist in colonization and invasion of extra-intestinal sites, causing it to be involved in urinary tract infections, meningitis, among other important diseases^51^. More worrying, in addition to the *ecp* operon, also known as fimbriae associated with meningitis and regulated by temperature (MAT), the MO strain carries the *aslA* gene, which has been shown to contribute to the invasion of the blood–brain barrier^52^.

The in vitro adherence assays showed that some bacterial strains (MO, TM2CRO, JM2CRO) were toxic to HeLa cells. In order to further analyze this phenotype, cytotoxicity assays on Vero cells were performed. Overall, the results indicated that all isolated strains are somewhat capable of damaging the host’s epithelia. Surprisingly, when Vero cells were cultivated with live bacteria, all strains showed a toxic effect, probably induced by a secreted substance rather than cell–cell contact, as pointed by the results obtained from incubation with dead bacterial cells. Therefore, we sought to test the effect of filtered supernatant. The TM2CRO strain resulted in cell damage, indicating that the observed cytotoxic effect was caused by a secreted toxin. Genome sequencing revealed that this strain harbors the *astA* gene that encodes the *E. coli* heat-stable toxin-1 (EAST-1). The presence of *astA* gene in *E. coli* strains has been reported worldwide, and it is commonly associated with diarrhea in humans^53,54^. However, when the boiled supernatant was tested, no cell damage was observed, leading to the conclusion that an unknown thermolabile toxin caused the cytotoxic effect. Interestingly, a vacuolating effect was observed in all ECMO treatments, indicating that this strain expresses a thermostable secreted toxin. Furthermore, genome sequencing revealed the presence of the *sat* gene, responsible for encoding the secreted autotransporter toxin (Sat). The cytopathic activity of Sat was reported on different epithelial cell lineages, such as HEp-2 and Vero cells^55,56^. This strain also carries the *senB* gene that encodes the secreted TieB enterotoxin. This toxin has been reported in different *Shigella* species and among other isolates of pathogenic and commensal *E. coli*, and plays a role in the development of severe diarrhea^57,58^.

Filtered supernatant of both *K. pneumoniae* strains also showed a cytotoxic effect. However, the damage was mainly on the cell nucleus. To determine if these secreted toxins were thermostable or thermolabile, boiled, filtered supernatants from JM2CRO and BO2 strains were tested, and nuclear damage to Vero cells was still observed, suggesting that these strains secrete a thermostable toxin, probably not yet identified. Interestingly, genes that code for a type 6 secretion system (T6SS) were detected in several strains evaluated in this study (C6O, 6M, TM2CRO, BO2, and JM2CRO). Although the primary role of T6SS is related to bacterial competition, the gene that encodes the effector protein Til1, detected in the BO2 strain, encodes a lipase that appears to have activity on eukaryotic cells^59,60^.

Evolutionary successful pathogenic bacterial lineages contain a plethora of virulence determinants that are essential for host colonization and pathogenesis. Among them, the iron/heme transport systems are crucial for colonization. All strains evaluated in this study carried at least one gene involved in iron acquisition, and those *E. coli* with ExPEC attributes (6M and MO) and both *Klebsiella* strains had more than 10 of these genes. The presence of different types of siderophores is an attribute of more virulent strains of *Klebsiella*^61^. This feature was also expected for ExPEC, since the concentration of iron in extra-intestinal sites is low, leading to the development of several strategies to sequester these molecules from the host^62^.

Another relevant aspect to be considered for the pathogenesis of bacterial strains isolated from seafood products investigated is the capacity to form biofilm, which was observed in all strains tested in this work. The ability to form biofilm can be considered an important virulence factor in gastrointestinal infections since it contributes to the colonization of the host by opportunistic pathogens and allows its persistence in the environment, where they can act as infectious agents for an extended period. In addition, life within the biofilm increases the resistance of the microorganism to the host's immune system and conventional antimicrobial drugs, making its eradication very difficult^63^. In fact, studies demonstrate that biofilm formation by pathogenic *E. coli* strains contributes to the persistence of the infection^64,65^, especially in strains that present an aggregative adhesion pattern, as observed in EM1CRO and C60 strains.

Although the most clinically significant *K. pneumoniae* biofilms are those formed on the internal surfaces of catheters and other internal devices, biofilm formation can also contribute to the colonization of the gastrointestinal tract, and the development of invasive infections, especially in immunocompromised patients^65^.

In summary, this study shed light on the emergence of critical-priority Enterobacterales co-harboring a broad repertoire of virulence and resistance factors isolated from wild-caught marine bivalves. Our findings are particularly worrisome because they suggest that wild-caught bivalves have been contaminated with medically important bacteria in the Southeast coast of Brazil, which reinforce the urgent need to strengthen surveillance of seafood sold in countries with the highest level of antimicrobial resistance. Additionally, our virulence assays confirmed the high pathogenic potential of the isolated bacteria, revealing a red-alert threat to seafood consumers. Special attention should be paid for uncooked seafood (*e.g.,* raw oysters), once the production and release of different toxins were documented in some strains. In this regard, the production of thermostable toxins is a worrisome prospect, as they would not be affected by cooking.

## Conclusion

We report the occurrence of critical-priority Enterobacterales in edible bivalves from a polluted area on the South America Atlantic coast. Using in vitro experiments, we also demonstrated the convergence of MDR phenotype with broad virulence repertoire that enables these bacteria to infect and harm human hosts, revealing a high pathogenic potential that can jeopardize the health of seafood consumers and marine-related ecosystems.

## Material and methods

### Sample collection and MDR bacterial isolation

Between November 2016 and February 2017, oysters (*Crassostrea* spp.) and brown mussels (*Perna perna*) samples were collected at low tide from 14 different locations at the marine coast of Sao Vicente (23.963056 S 46.391944 W) and Santos (23.960833 S 46.333361 W), two densely populated cities located on the southeast coast of Brazil. Each sample consisted of approximately ten mussels and ten oysters of similar sizes. The oyster and mussel samples were processed separately. The bivalves’ collection and processing were authorized by the appropriate Brazilian authority (SISBIO licenses n. 58,570–1). Bivalve samples were kept refrigerated in sterile plastic bags until processing (no later than 3 h after collection). Subsequently, 25 g of bivalves were incubated at 37 °C for 24 h in new sterile plastic bags (Whirl–Pak, Nasco, WI, USA) filled with 225 mL of Brain–Heart Infusion (BHI) broth. Following incubation, 1 mL of the BHI culture was inoculated onto MacConkey agar plates containing colistin (2 μg/mL), ceftriaxone (2 μg/mL) or meropenem (2 μg/mL), and incubated for an additional 24 h at 37 °C.

### Bacterial identification and antimicrobial susceptibility testing

All isolated Gram-negative bacterial colonies were identified by matrix-assisted laser desorption ionization-time of flight mass spectrometry (MALDI-TOF MS) analysis and tested for susceptibility to 15 different antimicrobials (amikacin, amoxicillin/clavulanic acid, aztreonam, cefepime, cefotaxime, cefoxitin, ceftazidime, ceftriaxone, ciprofloxacin, ertapenem, gentamicin, imipenem, meropenem, nalidixic acid, and sulfametoxazole-trimethoprim) by the Kirby-Bauer method, following the Clinical and Laboratory Standards Institute (CLSI) guidelines^67^. ESBL production was investigated using the double-disk synergy test (DDST)^67^ and minimum inhibitory concentrations (MICs) were determined using the E-test. The presence of genes encoding extended-spectrum β-lactamase (ESBL) (*bla*_CTX-M-1_, *bla*_CTX-M-2_, *bla*_CTX-M-8_ and *bla*_CTX-M-9_), cephamycin resistance (*bla*_CMY-2_), carbapenemase (*bla*_KPC-2_), and mobilized colistin resistance (*mcr-1*) was assessed by PCR. All PCR positive isolates were whole-genome sequenced.

### Whole-genome sequence analysis

Extraction of total bacterial DNA was performed using PureLink™ Genomic DNA mini Kit, and library preparation followed the Nextera XT Illumina protocol. Whole bacterial genome sequencing was performed using the Illumina NextSeq platform with paired-end reads (150-bp). De novo assembly was carried out using Velvet v.1.2.1 pipeline and Geneious R9 software*.* Serotypes, MLST, virulence genes, antimicrobial resistance genes, plasmid replicons, fimbriae type, and pMLST of *E. coli* isolates were identified using the SerotypeFinder 2.0, MLST 2.0, Virulence Finder 2.0, ResFinder 4.1, PlasmidFinder 2.1, FimTyper 1.0. and pMLST 2.0 databases available at the Center for Genomic Epidemiology—CGE (http://genomicepidemiology.org/), respectively. Phylogenetic groups of *E. coli* were determined in silico according to Clermont v1.4.0 (https://github.com/A-BN/ClermonTyping). For *K. pneumoniae*, Kleborate (https://github.com/katholt/Kleborate) was used for capsule (K) and wzi prediction, species confirmation, and multilocus sequence type (ST). CGE database was used to determine antimicrobial resistance, plasmid type, and pMLST. Additionally, ABRicate v0.9.8 (https://github.com/tseemann/abricate) with VFDB database (https://github.com/haruosuz/vfdb) was used to assess the presence of virulence genes and Kleborate v2.0.4 (https://github.com/katholt/Kleborate) was used for typing and to screen for mutations in quinolone resistance determining regions in *K. pneumoniae* strains. The presence of genes associated with resistance to heavy metal (*e.g.,* arsenic, lead, and mercury), disinfectants (*e.g.,* quaternary ammonium compounds), and pesticide (*e.g.,* glyphosate) was predicted by comparative in silico analysis against an in-house database. An identity threshold of > 98% was used to identify predicted genes. Genomic data were plotted on heatmaps for the presence/absence of virulence genes, resistance genes, and plasmids using iTOL v6 (https://itol.embl.de) with dummy trees.

### In vitro infection assays of HeLa cells

Before infection, HeLa cells were cultured in a 5% CO_2_ atmosphere for 48 h in 24-well plates (Nest, USA) containing DMEM medium (Cultilab Ltd.), and washed three times with phosphate-buffered saline (PBS). To each well, 960 µL of supplemented DMEM (*i.e.* 2% fetal bovine serum and 1% D-mannose) and 40 µL of the bacterial pre-cultures in Luria Bertani broth (LB) were added. The plates were incubated for 3 h or 6 h. Adherence assays were performed as described previously^68^. Briefly, cells were washed four times with PBS, fixed with methanol, and stained with May-Grünwald and Giemsa staining solutions (Merck, Germany) before analysis by light microscopy. The enteropathogenic *E. coli* strains E2348⁄69 (localized adhesion) and BA320 (localized-like adhesion) were used as positive controls. The ability of bacteria to invade epithelial cells was accessed as described previously^69^, with some modifications: after 3 h incubation, the extracellular bacteria were killed by the addition of fresh DMEM containing 100 µg/mL gentamycin or meropenem, and plates were incubated for additional 3 h at 37ºC. Cells were washed with PBS and lysed with 1% TritonX-100. Serial dilutions of the cell suspensions were plated on MacConkey agar. An enteroinvasive *E. coli* strain (O124:NM) and the non-invasive *E. coli* strain HB101 were used as positive and negative controls, respectively. Cell invasion was considered positive when bacterial growth on MacConkey agar plates was observed after 18 h incubation at 37ºC.

### In vitro cytotoxicity assay

VERO cells were cultivated in 24-well plates (Nest, USA) in DMEM medium (Cultilab Ltd.) containing 10% FBS, for 48 h at 37ºC, in a 5% CO_2_ atmosphere. The isolated strains were cultivated in LB broth for 18 h at 37ºC. An aliquot was centrifuged at 13,000 g for 2 min and the supernatant was filtered through 0.22 µm membrane filters (Millipore). Bacterial culture and supernatant filtrates aliquots were also boiled for 10 min. Fifty microliters of bacterial culture, supernatant, boiled bacterial culture or boiled supernatant were added to VERO cells, and after incubation for 18 h at 37ºC, the cells were washed three times with PBS, fixed with methanol and stained using May-Grünwald and Giemsa solutions. The results were compared with control cells. Cytotoxicity was considered positive when damaged cells were observed.

### Biofilm formation in microtitre plates (crystal violet assay)

The quantitative analysis of biofilm formation was carried out in 96-well polystyrene plates following previously described methodologies^70,71^, with slight modifications. Overnight bacterial cultures were inoculated into BHI broth at a dilution of 1:100 in 96-well polystyrene plates (TPP, Switzerland) in a final volume of 100 µL. After 24 h of incubation at 37 °C, the plates were washed 3 times with PBS to remove planktonic bacteria. The formed biofilms were fixed for 10 min at room temperature with 125 µL of a 75% ethanol solution and stained for 5 min with 125 µL of a 0.5% crystal violet solution. After being washed four times with PBS, the plates were left at room temperature to dry. Then, 200 µL of 30% glacial acetic acid were added and the plates were left for 2 min at room temperature to solubilize the crystal violet. Absorbance was read at 595 nm on the Multiskan®EX ELISA reader (Thermo Fisher Scientific, USA). All assays were performed in triplicates. Strains were considered to form weak, moderate, or strong biofilms, compared to a strong biofilm-forming *E.coli* strain (4157).

### Biofilm formation in glass slides and laser confocal microscopy

Microscopic analysis of biofilm formation was performed as described previously^65^. Briefly, overnight bacterial cultures were diluted 1:100 in fresh BHI broth and added to a 24-well cell culture plate with glass coverslips for a final volume of 1 mL. After 24 h of incubation at 37 °C and two PBS washes, the formed biofilms were fixed with 4% p-formaldehyde for 18 h at 4 °C, blocked for 30 min with a 0.2% bovine serum albumin solution, and permeabilized with 0.1% Triton X-100 for 5 min. After five PBS washes, the coverslips were removed from the plates, dried with filter paper, and fixed on glass slides with Mowiol (Calbiochem) added with Propidium Iodide (Molecular Probes) in a final concentration of 1:1000. After about 20 h at 4 °C, the slides were observed in a 630 × magnification confocal fluorescence microscope (Microscope LSM 510 Meta, Zeiss—Filters: BP500-530IR—488 nm; LP560—543 nm).

### Statistical analyses

Unless otherwise stated, all experiments were carried out in three independent triplicates. Statistical analyses were performed by comparison of means between groups using One-Way Anova (GraphPad Prism 9.0). The Post Hoc analysis was performed using the Tukey or Dunnett methods, according to the test. *p* < 0.0001 values were considered statistically significant.

## Data Availability

All data generated or used during this work are presented in the paper. The whole-genome nucleotide sequence of the bacterial strains used in this work are available in the GenBank database under the following access numbers: *E. coli* EM1CRO (NDBC00000000.1), *E. coli* C6O (NDBB00000000.1), *E. coli* 6M (NCWA00000000.1), *E. coli* MM (NDYX00000000.1), *E. coli* MO (NCVZ00000000.1), *E. coli* TM2CRO (NCVY00000000.1), *K. pneumoniae* BO2 (NCVX00000000.1), and *K. pneumoniae* JM2CRO (NCVW00000000.1).
